# Threonine 348 regulates the subcellular localization of PTEN

**DOI:** 10.1002/2211-5463.70328

**Published:** 2026-08-19

**Authors:** Takashi Kato, Suzu Tanaka, Miyu Ohashi

**Affiliations:** ^1^ Faculty of Pharmacy Yasuda Women's University Hiroshima Japan

**Keywords:** C2 domain, nucleus, plasma membrane, PTEN, subcellular localization, threonine 348

## Abstract

The tumor suppressor PTEN (phosphatase and tensin homolog) dephosphorylates PIP_3_ (phosphatidylinositol (3,4,5)‐trisphosphate) at the plasma membrane and protects genomic integrity in the nucleus; thus, regulation of PTEN subcellular localization is crucial. Previous studies have shown that the PTEN_350_ fragment is markedly enriched in the nucleus, a feature not explained by the N‐terminal nuclear localization signal. In this study, we generated PTEN fragments of various lengths and identified PTEN_348_ (residues 1–348), which showed prominent nuclear localization. Furthermore, the replacement of threonine 348 (Thr348) with other amino acids reduced the nuclear localization of the PTEN_348_ and PTEN_350_ fragments. Moreover, we found that PTEN_A4_ and PTEN_K13R,A4_ localized predominantly to the nucleus and plasma membrane, respectively, and that substitution of Thr348 with aspartic acid resulted in cytoplasmic localization in both mutants. Collectively, these results indicate that Thr348 is a key contributor to the regulation of PTEN subcellular localization.

## Graphical abstract

Thr348 in the C2 domain is a key contributor to PTEN subcellular localization. The PTEN350 fragment and PTENA4 accumulated in the nucleus, whereas PTEN_K13R,A4_ predominantly localized to the plasma membrane. In contrast, substitution of Thr348 with Asp (T348D) disrupted these characteristic localization patterns, resulting in predominant cytoplasmic localization.

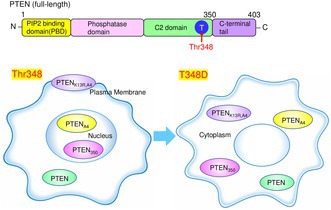

**Abbreviations**
AKT: protein kinase B (PKB)
CK2: casein kinase 2
DMEM: Dulbecco's modified Eagle Medium
FBS: fetal bovine serum
HEK293T: human embryonic kidney 293T
NES: nuclear export signal
NLS: nuclear localization signal
PIP2: phosphatidylinositol (4,5)‐bisphosphate
PIP3: phosphatidylinositol (3,4,5)‐trisphosphate
PTEN: phosphatase and tensin homolog
SUMO: small ubiquitin‐like modifier

PTEN (phosphatase and tensin homolog) is a lipid and protein phosphatase that functions as a tumor suppressor by dephosphorylating phosphatidylinositol (3,4,5)‐trisphosphate (PIP_3_) to phosphatidylinositol (4,5)‐bisphosphate (PIP_2_) at the plasma membrane, thereby negatively regulating the AKT (protein kinase B, PKB) signaling pathway [1, 2]. In addition to this well‐established role at the plasma membrane, PTEN has important nuclear functions, including the maintenance of genomic integrity [3, 4, 5]. Thus, regulation of PTEN subcellular localization is critical for its functional diversity.

PTEN consists of several distinct domains, including an N‐terminal PIP_2_‐binding motif, a phosphatase domain, a lipid‐binding C2 domain, a C‐terminal tail domain, and a PDZ‐binding motif [6, 7]. Two regions are critical for regulating PTEN subcellular localization. The first is lysine 13 (K13), whose mono‐ubiquitination is required for nuclear import [3, 8, 9]. Accordingly, substitution of K13 with arginine (K13R) prevented PTEN_K13R_ from entering the nucleus. The second region comprises four residues in the C‐terminal tail (Ser380/Thr382/Thr383/Ser385), whose phosphorylation promotes the cytoplasmic retention of PTEN [8, 10]. This C‐terminal region plays a critical role in regulating the conformation, stability, and activity of PTEN. Phosphorylation of these four residues promotes intramolecular interactions between the C‐terminal tail and the phosphatase–C2 core, thereby stabilizing the closed conformation of PTEN [11, 12, 13]. In contrast, the dephosphorylation of these four residues favors an open conformation that enhances membrane association. PTEN_A4_, in which these residues are substituted with alanine (A4), cannot be phosphorylated and therefore predominantly accumulates in the nucleus [8, 12, 13]. Structural transitions between the open and closed conformations can influence the accessibility of regulatory motifs and may also affect the subcellular trafficking of PTEN, including its nuclear import and export. In addition, the PTEN_351_ fragment, comprising residues 1–351, also shows strong nuclear localization [14]. The core domain of PTEN is generally considered to extend to residue 350 [15, 16]; therefore, PTEN_350_ was used as the core domain construct in this study. PTEN_350_ is markedly enriched in the nucleus. Although nuclear localization signals have been proposed at the N‐terminus, including K13 [17, 18], the mechanisms governing PTEN nuclear import remain unclear. For example, PTEN_32_, which consists of the first 32 residues of the N terminus, exhibits considerably lower nuclear localization than PTEN_350_. These findings suggest that additional elements are required for nuclear localization of the core domain.

In this study, we identified the essential regions and critical amino acid residues required for the nuclear localization of PTEN_350_. We also examined whether these key residues are necessary for the nuclear localization of PTEN_A4_. In addition, PTEN_K13R,A4_, a double mutant harboring both K13R and A4 substitutions, exhibits strong plasma membrane localization [8]. We further investigated whether these residues are necessary for the plasma membrane localization of PTEN_K13R,A4_.

## Results

### Threonine 348 is a critical residue for the nuclear localization of PTEN

**Fig. 1:**
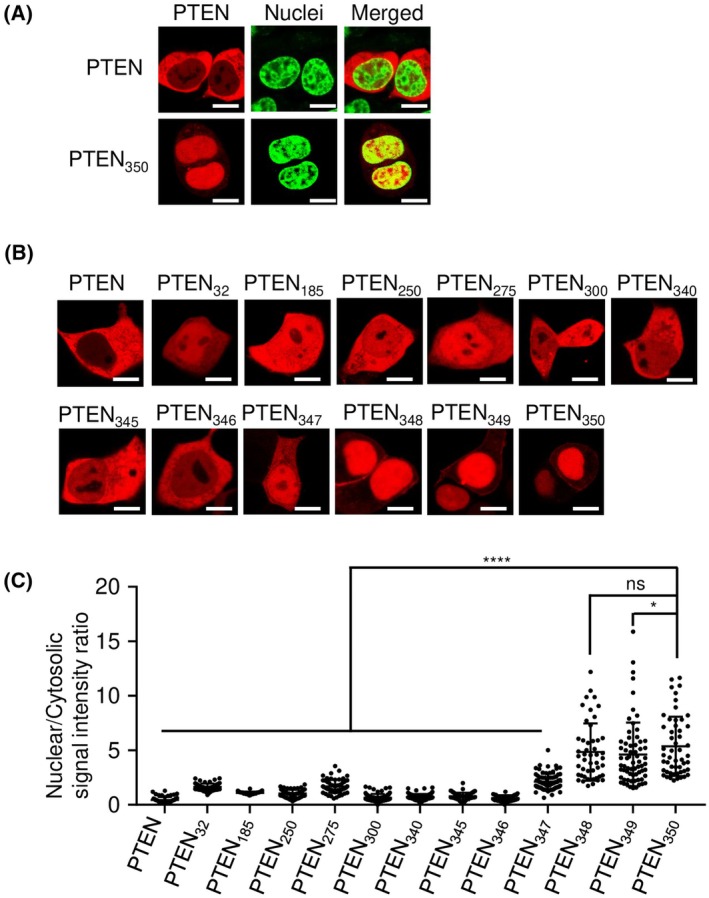
Threonine 348 is required for the nuclear translocation of PTEN_350_. (A) Full‐length phosphatase and tensin homolog (PTEN) and PTEN_350_ fused with mCherry were merged with Hoechst 33342 nuclear staining (B) Subcellular localization of PTEN fragments up to residue 350 in live HEK293T cells. (C) Quantification of the nuclear‐to‐cytoplasmic fluorescence intensity ratio of each PTEN fragment fused with mCherry. Data are presented as mean ± standard deviation (SD). (PTEN, *n* = 61; PTEN_32_, *n* = 61; PTEN_185_, *n* = 35; PTEN_250_, *n* = 64; PTEN_275_, *n* = 64; PTEN_300_, *n* = 76; PTEN_340_, *n* = 71; PTEN_345_, *n* = 55; PTEN_346_, *n* = 45; PTEN_347_, *n* = 80; PTEN_348_, *n* = 49; PTEN_349_, *n* = 66; PTEN_350_, *n* = 52 cells from three independent experiments). Representative images from three independent experiments are shown. Scale bar, 10 μm. One‐way analysis of variance (ANOVA) followed by Dunnett's test (vs PTEN_350_). *P < 0.05; ****P < 0.0001; ns, not significant.

PTEN and its fragments were fused to mCherry. Full‐length PTEN localized to both the cytoplasm and nucleus, with approximately twofold higher fluorescence intensity in the cytoplasm than in the nucleus (Fig. 1A). However, PTEN_350_ prominently accumulated in the nuclei of HEK293T cells (Fig. 1A). PTEN_32_ has been reported to localize to the nucleus via its N‐terminal signal [19]; however, its nuclear accumulation was markedly weaker than that of PTEN_350_ (Fig. 1B). These observations indicated that the nuclear localization of PTEN_350_ cannot be attributed solely to the N‐terminal nuclear localization signal, suggesting the presence of additional residues required for its nuclear localization. To determine whether other PTEN fragments exhibit similar localization patterns, we generated a series of truncation mutants, PTEN_185_, PTEN_250_, PTEN_275_, PTEN_300_, PTEN_340_, and PTEN_345_, in addition to PTEN_32_. These fragments were predominantly localized in the cytoplasm (Fig. 1B). Therefore, additional residues were sequentially added to PTEN_345_ to generate PTEN_346_–PTEN_349_. Although PTEN_346_ remained in the cytoplasm, PTEN_347_ showed increased nuclear accumulation. Importantly, PTEN_348_ was prominently localized in the nucleus (Fig. 1B). Statistical analysis revealed that the nuclear localization level of PTEN_348_ was not significantly different from that of PTEN_350_ (Fig. 1C).

PTEN_347_ exhibited increased nuclear accumulation; however, its nuclear localization level was markedly lower than that of PTEN_348_. These findings suggest that Phe347 may contribute to PTEN nuclear localization, whereas Thr348 appears to play a more prominent role in the predominant nuclear accumulation observed in PTEN_350_. Therefore, we focused on Thr348 and examined its contribution to nuclear localization.

### Effects of Thr348 substitutions on subcellular localization

**Fig. 2:**
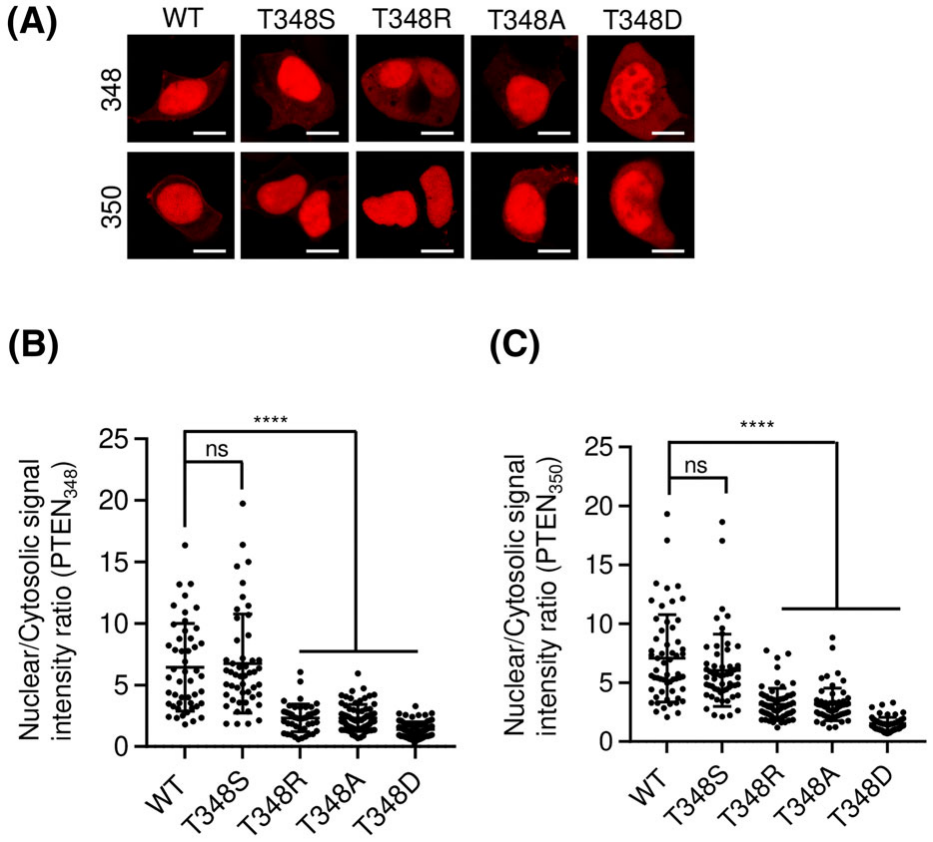
Subcellular localization of PTEN mutants with substitutions at threonine 348. (A) Subcellular localization of PTEN_348_ and PTEN_350_ mutants in live HEK293T cells. Representative images from three independent experiments are shown. Scale bar, 10 μm. (B) Quantification of the nuclear‐to‐cytoplasmic fluorescence intensity ratio of PTEN_348_ mutants fused with mCherry. Data are presented as mean ± SD. (wild‐type (WT), *n* = 48; T348S, *n* = 49; T348R, *n* = 46; T348A, *n* = 69; T348D, *n* = 74 cells from three independent experiments). (C) Quantification of the nuclear‐to‐cytoplasmic fluorescence intensity ratio of PTEN_350_ mutants fused with mCherry. Data are presented as mean ± SD. (WT, *n* = 57; T348S, *n* = 57; T348R, *n* = 66; T348A, *n* = 61; T348D, *n* = 69 cells from three independent experiments). Statistical analysis was performed using one‐way ANOVA followed by Dunnett's test against the corresponding wild‐type PTEN fragments in (B) and (C), respectively. ****P < 0.0001; ns, not significant.

To determine whether the role of Thr348 in nuclear localization depends on its chemical properties or its potential phosphorylation, we substituted Thr348 with serine, arginine, alanine, or aspartic acid in PTEN_348_ and PTEN_350_. Serine was used as a structurally similar substitute for threonine, whereas alanine was used as an uncharged substitute. In addition, aspartic acid was introduced as a negatively charged phosphomimetic residue, and arginine was introduced as a positively charged substitute. We first examined the localization of the T348S, T348A, T348R, and T348D mutants of PTEN_348_. While the T348S mutant was predominantly localized in the nucleus, the other mutants showed reduced nuclear accumulation (Fig. 2A). Quantification of the nuclear‐to‐cytoplasmic fluorescence intensity ratios confirmed a significant reduction in the nuclear localization of T348A, T348R, and T348D (Fig. 2B). Subsequently, the same mutations were introduced into PTEN_350_. Consistent with the results from the PTEN_348_ fragment, the T348S mutation had minimal or no effect on subcellular localization, whereas representative images showed reduced nuclear accumulation in the T348A, T348R, and T348D mutants (Fig. 2A). Notably, significant differences were observed between the serine/threonine group and the other substituted residues at this position (Fig. 2C).

### Thr348 is required for the nuclear localization of PTEN_A4_

**Fig. 3:**
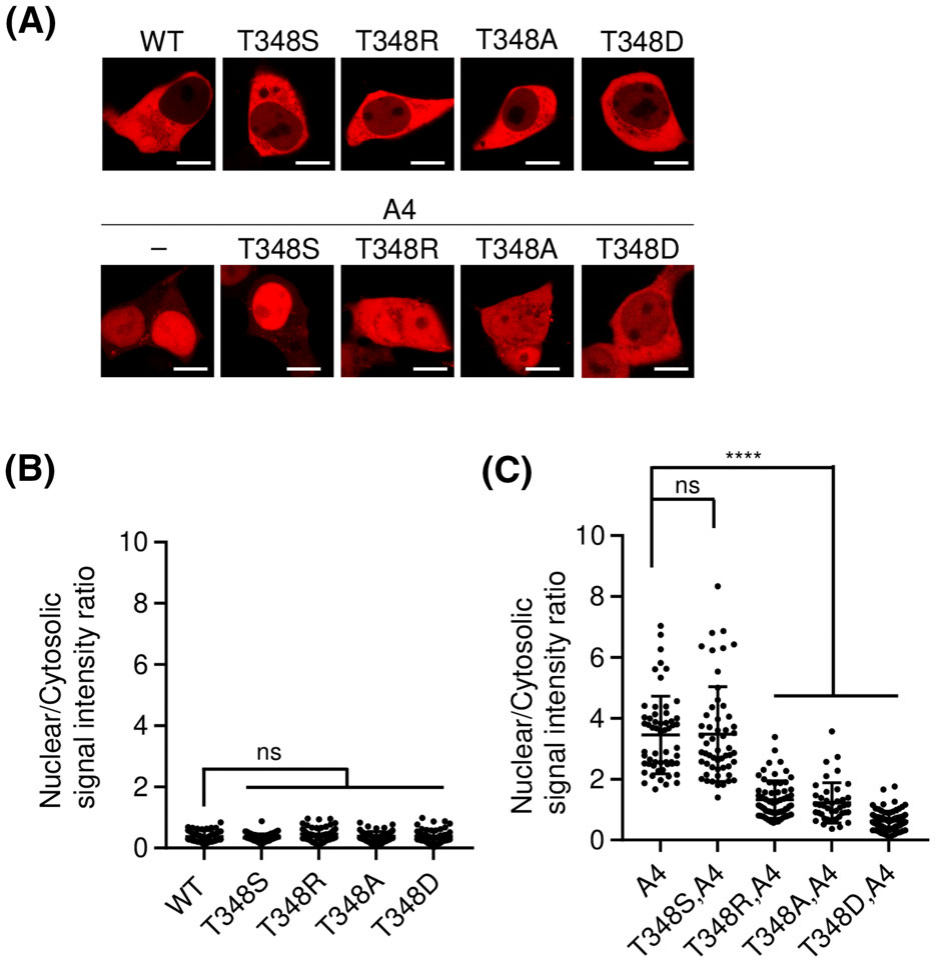
T348 substitution reduces the nuclear localization of full‐length PTEN and PTEN_A4_. (A) Subcellular localization of PTEN mutants in live HEK293T cells. Representative images from three independent experiments are shown. Scale bar, 10 μm. (B) Quantification of the nuclear‐to‐cytoplasmic fluorescence intensity ratio of PTEN mutants fused with mCherry. Data are presented as mean ± SD. (PTEN, *n* = 44; PTEN_T348S_, *n* = 63; PTEN_T348R_, *n* = 63; PTEN_T348A_, *n* = 77; PTEN_T348D_, *n* = 59 cells from three independent experiments). (C) Quantification of the nuclear‐to‐cytoplasmic fluorescence intensity ratio of PTEN_A4_ mutants fused with mCherry. Data are presented as mean ± SD. (PTEN_A4_, *n* = 57; PTEN_T348S,A4_, *n* = 64; PTEN_T348R,A4_, *n* = 63; PTEN_T348A,A4_, *n* = 42; PTEN_T348D,A4_, *n* = 93 cells from three independent experiments). – indicates that the mutation was not introduced. Statistical analysis was performed using one‐way ANOVA followed by Dunnett's test, with PTEN in (B) and PTEN_A4_ in (C), respectively. ****P < 0.0001; ns, not significant.

In the next set of experiments, we investigated the effects of these mutations (T348S, T348R, T348A, and T348D) on full‐length PTEN and PTEN_A4_. Initially, these mutations were introduced into PTEN, and their subcellular localization was examined. These mutations had no appreciable effect on the nuclear accumulation of wild‐type PTEN (Fig. 3A,B). Next, the effects of the Thr348 substitutions on the subcellular localization of PTEN_A4_, a variant previously shown to accumulate in the nucleus, were investigated. PTEN_A4_ has been reported to adopt an open conformation owing to disruption of intramolecular interactions between the C‐terminal tail and the phosphatase–C2 core [11, 14, 20]. Next, the T348S, T348R, T348A, and T348D mutations were introduced into PTEN_A4_, and their effects on its subcellular localization were examined. As expected, PTEN_T348S,A4_ showed nuclear localization similar to that of PTEN_A4_ (Fig. 3A). However, nuclear localization was reduced in PTEN_T348R,A4_ and PTEN_T348A,A4_, whereas PTEN_T348D,A4_ was predominantly localized in the cytoplasm rather than the nucleus. Quantitative analysis confirmed these observations, revealing significant differences in nuclear localization between the serine/threonine substitutions and the mutants carrying other substitutions at position 348 (Fig. 3C). These results suggest that Thr348 plays an important role in the nuclear localization of PTEN_A4_.

### Position 348 side‐chain chemistry contributes to plasma membrane localization of PTEN_K13R,A4_

**Fig. 4:**
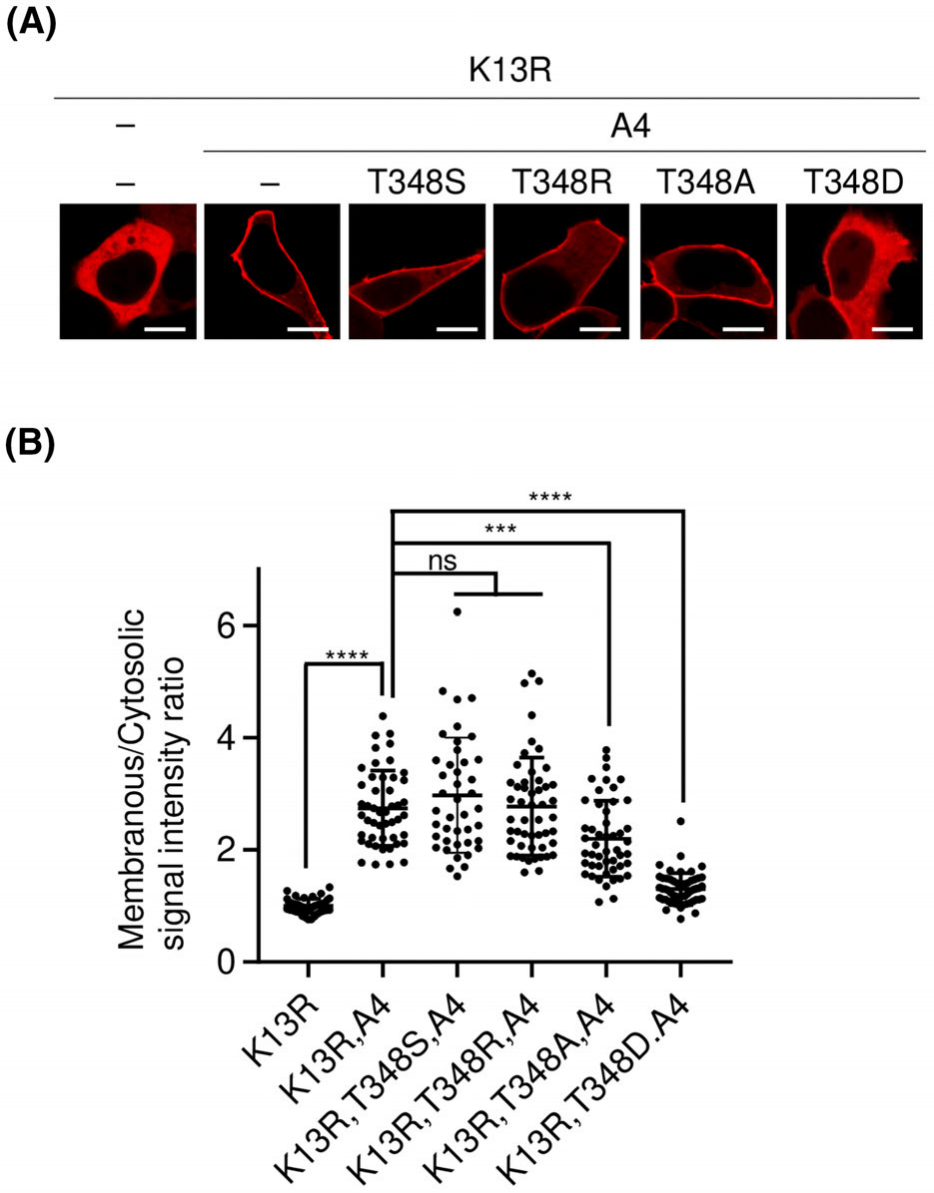
T348 substitution in PTEN_K13R,A4_ alters its localization to the plasma membrane. (A) Subcellular localization of PTEN mutants in live HEK293T cells. Representative images from three independent experiments are shown. Scale bar, 10 μm. (B) Quantification of the membrane‐to‐cytoplasmic fluorescence intensity ratio of PTEN mutants fused with mCherry. Data are presented as mean ± SD. (PTEN_K13R_, *n* = 43; PTEN_K13R,A4_, *n* = 48; PTEN_K13R,T348S,A4_, *n* = 42; PTEN_K13R,T348R,A4_, *n* = 50; PTEN_K13R,T348A,A4_, *n* = 49; PTEN_K13R,T348D,A4_, *n* = 54 cells from three independent experiments). – indicates that the mutation was not introduced. One‐way ANOVA followed by Dunnett's test (vs PTEN_K13R,A4_). ***P < 0.001, ****P < 0.0001; ns, not significant.

To determine whether Thr348 contributes to the formation of a conformation conducive to lipid binding, we investigated how Thr348 affects the plasma membrane localization of PTEN_K13R,A4_. PTEN_K13R,A4_ possesses an open C2 domain because of the A4 mutations, whereas the K13R mutation impairs nuclear localization, resulting in the preferential localization of PTEN_K13R,A4_ to the plasma membrane. Next, the T348 mutations were introduced into PTEN_K13R,A4_, and we assessed whether these variants retained plasma membrane localization. As shown in Fig. 4A, PTEN_K13R,A4_ and PTEN_K13R,T348S,A4_ predominantly localized to the plasma membrane. In contrast, PTEN_K13R,T348A,A4_ and PTEN_K13R,T348D,A4_ predominantly localized in the cytoplasm rather than at the plasma membrane. Quantitative analysis of the plasma membrane‐to‐cytoplasmic fluorescence intensity ratios demonstrated that PTEN_K13R,T348A,A4_ and PTEN_K13R,T348D,A4_ exhibited significantly reduced plasma membrane localization compared with PTEN_K13R,A4_ (Fig. 4B). Taken together, these findings suggest that the presence of a hydroxyl group at position 348 contributes to plasma membrane localization of PTEN_K13R,A4_ and may facilitate its association with membrane lipids.

## Discussion

In the present study, we demonstrated that Thr348 represents a key regulatory element involved in the control of PTEN nuclear localization. Thr348 is located within the C2 domain, the principal lipid‐binding module [21, 22]. Given the importance of PTEN–lipid interactions in membrane association, we hypothesized that Thr348 contributes to the association of PTEN_350_ and PTEN_A4_ with nuclear membrane lipids. Consistent with this hypothesis, the T348D substitution markedly diminished the plasma membrane localization of PTEN_K13R,A4_, suggesting that Thr348 is important for membrane association. In contrast, the full‐length PTEN adopts a closed conformation via intramolecular interactions between its C‐terminal tail and C2 domains. This conformation is likely to mask Thr348, thereby limiting its membrane association and contributing to the diffuse intracellular distribution of wild‐type PTEN. Given that the nuclear envelope is a lipid bilayer, the effect of Thr348 on membrane association may be related to its nuclear accumulation. Consistent with this possibility, the substitution of Thr348 with aspartic acid or arginine significantly impaired the nuclear accumulation of PTEN_350_. These findings suggest that Thr348‐mediated membrane association contributes not only to plasma membrane localization but also to nuclear localization.

Nuclear transport of PTEN is critical for its tumor‐suppressive functions and has attracted considerable interest from a mechanistic perspective, given the central role of PTEN as a lipid and protein phosphatase. Importin‐11 has been identified as a nuclear import receptor for PTEN, and this process requires the ubiquitin‐conjugated enzyme UBE2E1 [23]. However, the molecular features of PTEN that facilitate its recognition by the nuclear import machinery remain poorly understood. Our findings suggest that Thr348 may represent one such determinant by promoting membrane interactions that facilitate nuclear localization. Future studies aimed at defining the structural and biochemical basis of Thr348‐dependent membrane association, as well as its contribution to PTEN–lipid interactions, will provide further insights into the mechanisms governing PTEN nuclear transport.

PTEN contains multiple phosphorylation sites, among which those in the C‐terminal tail—Ser380, Thr382, Thr383, and Ser385—have received particular attention. The phosphorylation of these residues by casein kinase 2 is essential for maintaining the closed conformation of PTEN. Consequently, PTEN becomes less accessible to E3 ubiquitin ligases, enabling it to evade proteasomal degradation [24]. Casein kinase 2 also phosphorylates Ser370, which promotes the phosphorylation of Ser362 and Thr366 by glycogen synthase kinase 3. Although these modifications have been implicated in the stability and cytoplasmic distribution of PTEN, their precise functional significance remains unclear [25]. Phosphorylation within the C2 domain has also been reported. In particular, phosphorylation of Tyr240 and Tyr315 by Src kinase induces protein instability and diminishes PTEN's affinity for the plasma membrane [25]. However, the underlying mechanisms and the associated conformational changes remain unclear [26]. Whether Thr348 is phosphorylated and, if so, whether its phosphorylation correlates with conformational transitions, subcellular distribution, or PTEN function remains unclear. Future studies aimed at identifying the kinase(s) responsible for Thr348 phosphorylation and determining how this modification influences PTEN conformation, membrane association, and nuclear transport are crucial for understanding the mechanisms regulating PTEN activity and its tumor‐suppressive functions.

Since nuclear accumulation may reflect alterations in either nuclear import or export, we cannot distinguish between these mechanisms based solely on localization data. Although computational analysis did not identify a canonical NES near Thr348 and previous reviews have not reported a well‐defined export signal in this region [27], we cannot formally exclude the possibility that Thr348 influences nuclear export through indirect or non‐canonical mechanisms.

Alternatively, the observed effects of Thr348 substitutions on nuclear accumulation may reflect altered nuclear import efficiency. Whether Thr348 primarily affects nuclear import, nuclear export, or both remains to be determined.

In the present study, we demonstrated the importance of Thr348 in the nuclear localization of PTEN. However, several questions remained unanswered. Notably, PTEN_347_ retained partial nuclear localization despite the loss of Thr348, suggesting that Phe347 may also contribute to PTEN nuclear localization. Future studies are needed to determine the functional contribution of Phe347 and to clarify its relationship with Thr348 in regulating PTEN nuclear localization. In addition, although the T348D substitution significantly reduced the nuclear accumulation of PTEN_350_, detectable nuclear localization was still observed for PTEN_350,T348D_. Although the T348D substitution significantly reduced the nuclear accumulation of PTEN_350_, detectable nuclear localization was still observed for PTEN_350,T348D_. The mechanism underlying this residual nuclear localization remains unclear and warrants further investigation.

Notably, the addition of the C‐terminal tail to PTEN_350,T348D_ resulted in predominantly cytoplasmic localization, a phenomenon observed not only in PTEN but also in PTEN_A4_. These findings suggest that the C‐terminal tail contains elements that promote cytoplasmic localization. This effect was retained even in PTEN_A4_, in which the canonical Ser380/Thr382/Thr383/Ser385 phosphorylation motif was disrupted. Whether this phenomenon reflects a size‐dependent effect of the C‐terminal tail or the presence of specific regulatory sequences outside the Ser380/Thr382/Thr383/Ser385 motif remains to be determined.

A slight increase in nuclear localization was observed in the PTEN_275_ fragment compared to that in PTEN_250_. This modest increase may reflect the influence of additional regulatory elements in this region. Notably, the two major SUMOylation sites, K254 and K266, are located between residues 250 and 275 [7, 28]. SUMOylation at K254 impairs nuclear export in DNA‐damaged cells [29], whereas SUMOylation at K266 promotes the association between PTEN and the plasma membrane via electrostatic interactions [30]. Therefore, these modifications may have contributed to the enhanced nuclear accumulation observed in PTEN_275_. Because the present study was conducted under non‐stressed conditions, only a minor increase was detected; however, more pronounced nuclear accumulation might be expected under conditions of DNA damage.

The subcellular localization of PTEN has been extensively characterized, and its significance in diverse cellular functions has become increasingly evident. Given the distinct roles of PTEN in the nucleus, cytoplasm, and plasma membrane, elucidating the molecular mechanisms governing its subcellular localization is of critical importance. In addition to the established mechanisms regulating PTEN localization, our findings identify Thr348 within the C2 domain as an important contributor to nuclear translocation of PTEN. Further mechanistic insights into how this residue participates in the regulatory network controlling PTEN localization will be essential to define its broader biological significance.

## Materials and methods

### Plasmids

The pmCherry‐N1 plasmid was provided by Dr. Nakamura (Kyoto University) [31, 32]. Polymerase chain reaction (PCR)‐amplified PTEN fragments and their mutants were cloned into the pmCherry‐N1 vector to generate the corresponding constructs. PCR was performed using ExPremier DNA Polymerase (Takara Bio, Shiga, Japan), and all constructs were verified by DNA sequencing (Eurofins Genomics, Tokyo, Japan). Restriction enzymes and T4 DNA ligase were purchased from Nippon Gene (Toyama, Japan) and New England Biolabs (M0202; Ipswich, MA, USA), respectively.

### Cell culture and transfection

Human embryonic kidney 293T (HEK293T; RRID:CVCL_0063) cells were kindly provided by Dr. Nonaka [33] and maintained in Dulbecco's modified Eagle's medium (DMEM; 043‐30085, FUJIFILM Wako Pure Chemical, Osaka, Japan) supplemented with 10% fetal bovine serum (A525671; Gibco, Grand Island, NY, USA). The HEK293T cells were authenticated by short tandem repeat (STR) profiling through Promega Corporation (Madison, WI, USA). The obtained STR profile was consistent with reference HEK293T cells profiles registered in the JCRB Cell Bank and ATCC databases. Mycoplasma contamination was assessed by microscopic observation and PCR‐based Mycoplasma Detection Set (6601; Takara Bio). No evidence of mycoplasma contamination was detected. Cells were transfected with plasmid constructs using Lipofectamine 3000 (L3000015; Invitrogen, Carlsbad, CA, USA) according to the manufacturer's instructions.

### Live cell imaging

The day after transfection, samples were imaged using an Olympus FV1000 laser‐scanning confocal microscope equipped with a 60× oil‐immersion objective lens (NA 1.35) and fv1000‐asw software (Olympus, Tokyo, Japan). Nuclei were stained with Hoechst 33342 (Dojindo, Kumamoto, Japan). All imaging experiments were independently repeated three times with similar results. Representative images are shown in the figures.

### Signal quantification and statistical analysis

Quantification of fluorescent signals was performed using fiji (imagej; NIH, Bethesda, MD, USA). Nuclear and cytosolic fluorescence intensities were quantified as the mean fluorescence intensity within manually defined nuclear and cytosolic regions of interest, respectively. Background fluorescence was confirmed to be negligible (approximately zero); therefore, no background subtraction was performed. To measure the signal intensity along the plasma membrane, a line was drawn across individual cells, and the fluorescence intensity along the line was quantified using the Plot Profile function in fiji. The regions corresponding to the plasma membrane and cytoplasm were defined based on the intensity profile, and the membrane‐to‐cytoplasm ratio was calculated. The analysis workflow is shown in Fig. S1.

Statistical analyses were performed using graphpad prism 10 software (GraphPad Software, Inc.).

## Conflict of interest

The authors declare no conflict of interest.

## Author contributions

TK was responsible for study conception and design, experimental performance, analysis and interpretation of the results, and manuscript preparation. ST and MO performed the experiments and contributed to the data analysis and interpretation.

## Supporting information


**Fig. S1.** Subcellular localization of each PTEN mutant was quantified by measuring fluorescence intensity at the plasma membrane and in the cytosol.

## Data Availability

Data generated in this study is available from the corresponding author upon request.
