# Supplementary material for: Threonine 348 regulates the subcellular localization of PTEN
Source: FEBS Open Bio. 2026 Aug 19:10.1002/2211-5463.70328. Online ahead of print. doi: 10.1002/2211-5463.70328 (PMC13487556; doi:10.1002/2211-5463.70328)
Supplement: Supplementary file 1 [file FEB4-9999-0-s001.pdf]

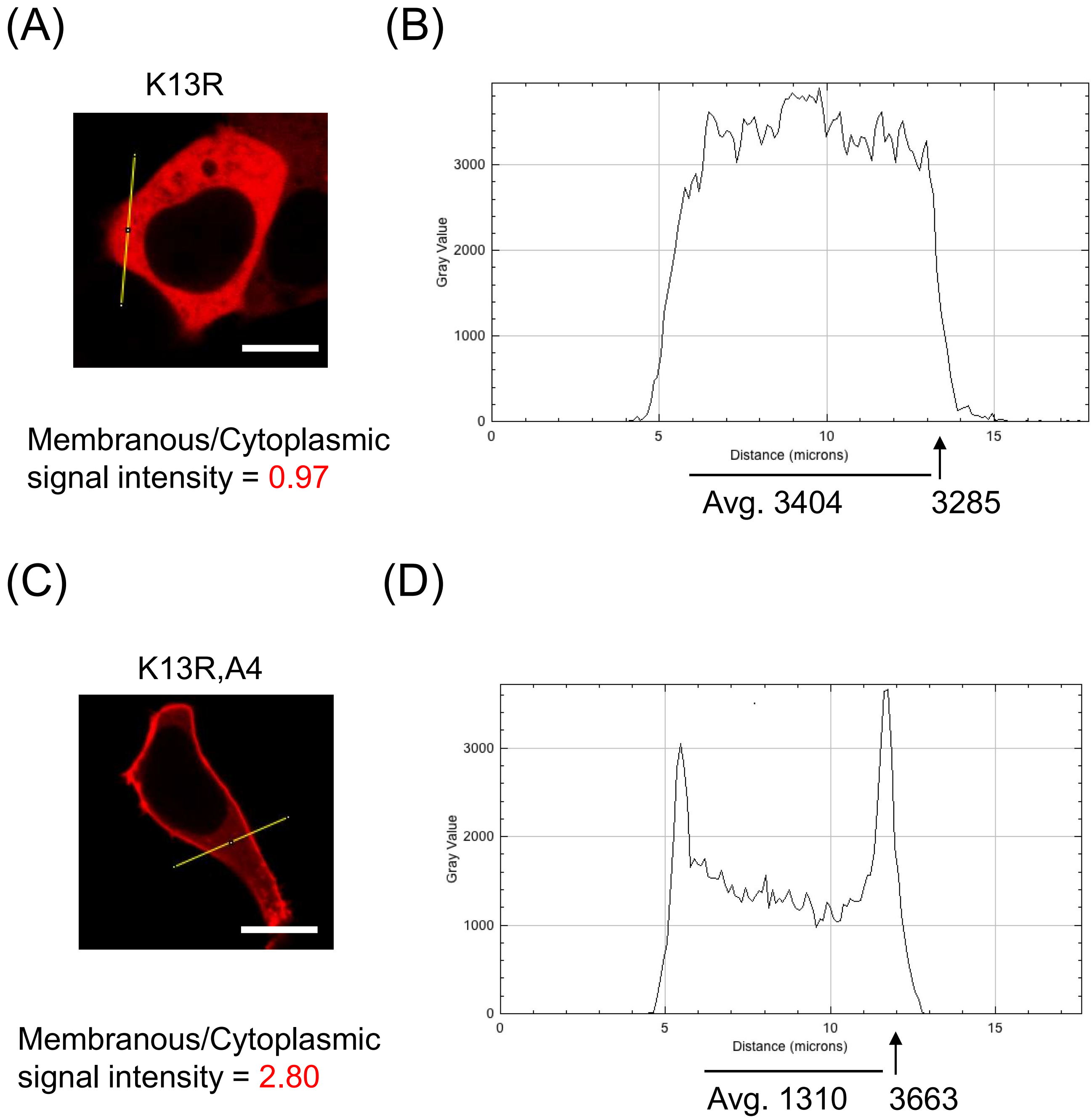

Fig. S1. Subcellular localization of each PTEN mutant was quantified by measuring fluorescence intensity at the plasma membrane and in the cytosol. (A, C) A line was drawn across individual cells using Fiji (ImageJ) software. (B, D) Fluorescence intensity profiles were obtained along the line. The peak fluorescence intensity at the plasma membrane and the mean fluorescence intensity of the adjacent cytosolic region were measured. Scale bar, 10 µm. (A, B) $PTEN_{K13R}$. (C, D) $PTEN_{K13R,A4}$.
